# Estimating the number of people living with dementia at different stages of the condition in India: A Delphi process

**DOI:** 10.1177/14713012231181627

**Published:** 2023-06-05

**Authors:** Nicolas Farina, Jayeeta Rajagopalan, Suvarna Alladi, Aliaa Ibnidris, Cleusa P Ferri, Martin Knapp, Adelina Comas-Herrera

**Affiliations:** Faculty of Health, 6633 University of Plymouth, Plymouth, UK; Strengthening Responses to Dementia in Developing Countries (STRiDE) India, 29148 National Institute of Mental Health and Neuro-Sciences (NIMHANS), Bangalore, India; Department of Psychiatry and Mental Health, 37716 University of Cape Town, South Africa; Department of Psychiatry, School of Medicine, 28105 Universidade Federal de São Paulo, Sao Paulo, Brazil; 4905 London School of Economics and Political Science, London, UK

**Keywords:** alzheimer’s disease, diagnosis, severity, consensus, delphi

## Abstract

**Introduction:**

Numerous studies have previously estimated the dementia prevalence in India. However, as these estimates use different methodologies and sampling strategies, generating definitive prevalence estimates can be difficult.

**Methods:**

A Delphi process involving eight clinical and academic experts provided prevalence estimates of dementia within India, split by sex and age. The experts were also asked to estimate the number of people potentially living at different stages of the condition. A priori criteria were used to ascertain the point in which consensus was achieved.

**Results:**

Our consensus estimates generated a dementia prevalence of 2.8% (95% CI = 1.9 to 3.6) for those aged 60 years and above in India. Consensus was achieved across age and sex prevalence estimates, with the exception of one (females aged 60–64). Our experts estimated that 42.9% of people living with dementia in India had a mild severity.

**Conclusions:**

The findings indicate that there could be approximately 3.9 million people living with dementia in India, of which 1.7 million could be living with dementia of mild severity. Such estimates can better help researchers and policy makers to estimate the true cost and impact of dementia in India and can inform resource allocation decisions.

## Introduction

Population projections estimate that there are currently nearly 138 million older adults (aged 60+) in India, this is set to rise to almost 194 million by 2031 (National Commission on Population, 2020). Dementia is a condition that disproportionately affects older adults, leading to cognitive and functional decline. Estimating the numbers of people with dementia within a given country is essential to better understand the impact of dementia on a societal level. Within India, prevalence studies of dementia have been conducted across different regions of the country (e.g. Banerjee et al., 2017; Chandra et al., 1998; Das et al., 2006, 2008; Mathuranath et al., 2010; Poddar et al., 2011; Raina et al., 2008, 2014, 2016; Rajkumar et al., 1997; Razdan et al., 1994; Rodriguez et al., 2008; Saldanha et al., 2010; Seby et al., 2011; Shaji et al., 2005; Vas et al., 2001) with varying estimates generated. These differences in estimates may be attributed to variability in sociodemographic, cultural, genetic and environmental factors in addition to varying methodological approaches adopted by individual studies (Das et al., 2012). Through the use of statistical modelling, the Global Burden of Disease [GBD] India study estimated 3.69 million (95% UI 3.13–4.25) people were living with Alzheimer’s disease and other dementias in India in 2019 (Singh et al., 2021).

Meta-analysis can assist in synthesising and compiling these data from individual studies. Pooled prevalence estimates for dementia in India have ranged from 3.4% (95% CI = 2.0 to 5.0) to 4.4% (2.2–7.2) (Dhiman et al., 2021; Farina et al, 2020). However, a major factor to consider is that estimates are dependent on review inclusion criteria, which may be influenced by a whole range of methodological heterogeneity, most notably diagnostic criteria. For example, prevalence estimates have been found to be lower in those utilising DSM-IV diagnostic criteria in comparison to those using other diagnostic algorithms such as 10/66 diagnostic schedule (Farina et al., 2020; Paddick et al., 2013). This may be because the DSM-IV criteria are missing milder cases of dementia, or that the 10/66 algorithm is over-identifying cases of dementia (Paddick et al., 2013; Rodriguez et al., 2008). As a result of differences between prevalence estimates and individual study reporting styles, it can be difficult for systematic reviews to extract data to obtain more granular estimates (e.g., age, sex) in a meta-analysis. For example, very few systematic reviews (e.g., Choudhary et al., 2021) from India have provided prevalence of dementia split by age and sex, despite individual studies reporting on them. In addition, there are also considerable gaps in the existing evidence base, which makes it difficult to compile such data. A Delphi process is one approach that can help overcome such gaps, through the use of experts to critically generate estimates using best evidence, whilst being able to draw from their expertise. Such approaches have been previously used to estimate dementia prevalence globally (e.g., Ferri et al., 2005).

Dementia is largely underdiagnosed, with only 1 in 10 persons with dementia receiving a diagnosis, treatment or care in India (Nulkar et al., 2019). Lack of awareness, stigma associated with the condition, shortage of resources such as health infrastructure and specialists to diagnose dementia contribute to this gap (Alzheimer’s and Related Disorders Society of India, 2010). Consequently, it is not possible to ascertain the true number of people living at different stages of the condition in India based on clinical records alone. Efforts have been conducted to improve diagnosis rates in a country with such educational and linguistic diversity. Culturally appropriate tools such as the Indian Council of Medical Research-Neurocognitive Toolbox (ICMR-NCTB) (Iyer et al., 2020; Verma et al., 2021) have been developed and validated to aid in the establishment of accurate dementia diagnosis and prevalence estimates.

Care has a cost, and care needs will inevitably grow at an individual level as dementia progresses and impairment increases. For example, it has previously been estimated that care (e.g., formal care, informal care and medical costs) costs 45,600 Indian Rupees per year for a person with mild dementia (in an urban setting), whilst it costs over 2,02,450 Indian Rupees a year for a person with severe dementia (Rao & Bharath, 2013). The increased cost of care between severity was driven in part by increased residential care. The authors also report a lower cost of care in rural settings, which could be attributed to a lack of access to institutional care outside of metropolitan areas (Alzheimer’s and Related Disorders Society of India, 2010). Irrespective, informal care typically is the largest cost for people with dementia in low- and middle- income settings, more so than social sector costs and direct medical costs (Wimo et al., 2018). There is much needed to understand the associated costs and impact of dementia in India to facilitate appropriate resource allocation and planning of service delivery.

This study aimed to generate and achieve expert consensus on:

(1) The dementia prevalence for those aged 60 years and older in India, split by sex and age.

(2) The percentage of people living with mild, moderate and severe dementia in India.

## Methods

We adopted a methodology similar to the one adopted in a global prevalence of dementia study (Ferri et al., 2005). Unlike a traditional Delphi study, we aimed to achieve consensus on estimates of dementia prevalence and dementia severity split through a series of unbound integer responses (i.e., expert provides numeric estimates, rather than being asked to rate their agreement or confidence on a specific estimates).

Eight experts were selected by a senior Indian neurologist and co-author (SA) on the basis of their experience and knowledge of the dementia field in the country. There is no consensus on the number of experts required for a Delphi study. For example, one study recommended that ten to 15 experts is considered sufficient, though it should be acknowledged that smaller numbers do minimise potential logistical issues of running the Delphi technique (see Nashir et al., 2015). As per existing guidance (Adler & Ziglio, 1996; Skulmoski et al., 2007), experts were required to:

(i) have the appropriate knowledge and experience,

(ii) be willing to participate,

(iii) have sufficient time (to participate) and,

(iv) possess effective communication skills.

We prioritised experts with experience of research associated with estimating dementia prevalence within India. These experts were selected through snowballing techniques and utilising existing networks. Those selected as experts were not involved in the design, set up, or analysis involved in the Delphi study.

Each expert was sent an email from co-author (JR) describing the Delphi process and how their information/contributions would be treated if they decided to participate. All experts were informed that their individual responses would remain anonymised, though their involvement in the process would be acknowledged in any write-up (if they so wished).

In the first round, experts were provided a summary of evidence of prevalence data derived from the country (as identified in a previous publication; Farina et al., 2020). These prevalence data consisted of methodological details (setting, sample size, one-phase or two-phase survey, implementation of two-phase design, response rates for both phases, diagnostic criteria) and we summarised specific prevalence estimates from every study. We made no judgement about the quality or risk of bias of each study. The experts were explicitly informed that they do not need to use the prevalence data compiled. In a pre-designed form on REDCap (Harris et al., 2009, 2019) hosted at the London School of Economics and Political Science, participants were asked to estimate the prevalence of dementia based upon their knowledge and/or using the information provided. These fields were split by age and sex. A minimum age of 60 years old was used to reflect that the vast majority of dementia cases occur in old age, whilst also aligning with many epidemiological studies that estimate dementia prevalence within India. Age categories were based on the available population demography from India (i.e., 60–64, 65–69, 70–74, 75–79, 80+).

Experts were also asked to provide an estimate of the percentage of people living with dementia in India, categorised by severity. We followed The Longitudinal Ageing Study in India (LASI; Perianayagam et al., 2022) protocol that calculated the percentage in the lowest 10th percentile of the composite cognition score, as this was identified “as a proxy measure of poor cognitive functioning” (International Institute for Population Sciences, 2020, pp.374). From the composite cognition score we excluded serial 7s and backward counting from the original arithmetic domain due to missing values and to avoid our score being more relevant to measure education attainment than cognitive functioning respectively. After this, we operationalised the definition of severity of impairment by combining 10th percentile of the composite cognition score with Activities of Daily Living [ADL] (difficulty in walking across the room, bathing, eating, dressing, getting in or out of bed, or toilet use) as measured in the LASI dataset. This is in line with previous methodology (Comas-Herrera et al., 2007). The experts were provided the following guidance on how we defined severity using the LASI dataset:

(1) Mild impairment: 10th percentile of composite cognition score and 1 ADL

(2) Moderate impairment: 10th percentile of composite cognition score and 2 ADL

(3) Severe impairment: 10th percentile of composite cognition score and 3 plus ADL

Even if experts were unsure, they were asked to be provide their best estimate. Experts had the opportunity to add comments to support why they believed their estimates to be accurate. Alongside each set of responses, the experts were asked to self-report their confidence in their estimate (see Adler & Ziglio, 1996). Once all responses were received, data were compiled and summarised.

In the second round, experts were provided with a summary of evidence from the first round and were asked to estimate the prevalence in fields that did not reach consensus (see definition below). In this round, experts were provided information about the average responses (mean), and how their revised scores compared to the average response in the previous round. In addition, a summary of anonymised comments alongside confidence ratings were provided. Experts were informed that they did not have to change their estimates if they did not want to.

It was decided that the Delphi process would stop once a) consensus was achieved across all fields, b) we had less than 70% response rates following a given round (thus affecting the study’s validity), c) following three rounds irrespective of whether consensus had been achieved across all fields.

In this study we did not do a third round because the response rate dropped below 70% in the second round.

### Achieving consensus

Consensus was determined by using an established a priori statistical criteria used in previous Delphi studies (von der Gracht, 2012). For consensus to be achieved in a given field following each round, the experts pooled estimates require to: a) achieve an Interquartile range (IQR) < 1, or b) the co-efficient of variation (CoV) ≤ .5. The choice to utilise both criteria ensured that consensus could be achieved even in situations where estimate values were high, or where there were a small number of extreme outliers. The mean estimate for each field were extracted and used as the value that represented consensus to accommodate inevitable variations in estimates.

### Analysis and reporting

Findings were summarised narratively for each round, with fields being grouped together based on theme (i.e., prevalence split by age, dementia severity) with an emphasis on fields that achieved a consensus. Fields that did not achieve consensus were also discussed, and the implications of this were considered. For each round and response, a mean, median, standard deviation, CoV and IQR were calculated and reported. Summary statistics of experts’ confidence in their estimates were also reported in round one.

### Missing data

During each round, we followed-up with experts if they did not respond or there were any gaps in the responses provided. If experts did not respond after three emails, then they were classed as drop-out.

### Maintaining anonymity

The researcher (NF) who developed the forms and analysed data, did not have access to the names of the experts who responded to each round. A second researcher, who was not involved in the analysis process (JR), was responsible for liaising with the experts via email. All emails were sent out individually or blinded to other experts. In instances where the expert provided comments which could have revealed their identity, minor changes were made to maintain anonymity (e.g., “…based on our previous research (citation)” to “…based on previous research (citation)”.

### Ethical approvals

All experts provided informed consent to share their expertise as part of the Delphi process. As this manuscript involves expert opinion rather than research on human subjects, we did not obtain ethical approval.

## Results

### Round 1: Overview

Of the 13 experts initially invited, eight expert responses were received. See Supplementary Table 1 for a summary of expertise.

#### Prevalence split by sex and age

Individual experts’ estimates ranged from 0.5% (prevalence of males aged 60–64) to 27% (prevalence of males aged 80 years and over). Irrespective of sex, the average estimate increased by age category. Variability of estimates remained quite high, with no field meeting the a priori threshold of consensus either based on the IQR or CoV. See Table 1.

**Table 1.** Average estimate of dementia prevalence in India split by age and sex after the first round with experts (n = 8).

Age	Sex	Mean	SD	Mdn	IQR	CoV	Meet *a priori* consensus
60–64	Male	2.6	3.2	1.5	2.0	1.2	No
	Female	2.0	1.5	1.6	3.1	0.8	No
65–69	Male	3.9	4.8	2.3	3.9	1.2	No
	Female	3.8	3.6	2.5	3.8	1.0	No
70–74	Male	6.0	6.6	3.3	4.5	1.1	No
	Female	5.5	4.8	3.8	6.5	0.9	No
75–79	Male	7.7	6.2	4.8	5.6	0.8	No
	Female	8.0	6.2	5.2	6.9	0.8	No
80+	Male	11.9	7.8	9.6	10.5	0.7	No
	Female	12.2	7.1	10.2	10.7	0.6	No

#### Total prevalence

Overall based on round 1 estimates, dementia prevalence was 4.9% for those aged 60 years and over in India, with a prevalence of 5.0% for males and 4.8% for females. Five of the experts were confident with their responses (60%–79% confident of being right), two were very confident (80%–99% confident of being right), whilst one expert was less so (40%–59% confident of being right).

#### Severity

A single expert’s data entry had to be excluded because of a typographical error, leaving seven experts providing estimates on severity. Experts on average estimated that the largest proportion of people will be living with mild dementia (42.9%), followed by moderate (31.9%) and severe (25.2%). The mild and moderate severity estimates met the CoV threshold for consensus, though the severe estimate did not. See Table 2 for further details. Three experts were very confident with their estimates (80%–99% confident of being right), two experts were confident (60%–79% confident of being right), one expert was unsure (40%–59% confident of being right), and a single expert did not provide a confidence rating.

**Table 2.** Estimate of the percentage of people living with dementia in India split by severity (n = 7).

	Mean	SD	Mdn	IQR	CoV	Meet *a priori* consensus
Mild	42.9	21.5	40	45.0	0.5	Yes
Moderate	31.9	10.8	30	17.5	0.3	Yes
Severe	25.2	15.6	25	27.5	0.6	No

### Round 2: Overview

Between rounds three experts dropped out (non-response), thus leaving five experts for round two. Of the three experts that dropped out between rounds, two were estimating higher than average prevalence in first round (i.e., >5.0% for total male prevalence and 4.8% for total female prevalence, respectively).

#### Prevalence split by sex and age

In round two, consensus was achieved across experts on male prevalence split by age, ranging from 0.9% in the 60–64-year-old category to 9.4% in the 80 years and older category. For female estimates all estimates reached consensus apart from the 60–64-year-old group. Consistently for each age category, females were estimated to have an equal or higher dementia prevalence than males. Table 3 presents full details about average estimates and variability.

**Table 3.** Average estimate of dementia prevalence (%) in India split by age and sex after the second round with experts (n = 5).

Age	Sex	Mean	SD	Mdn	IQR	CoV	Meet *a priori* consensus
60–64	Male	0.9	0.5	0.6	0.5	0.6	Yes
	Female	0.9	0.5	0.8	1.1	0.6	No
65–69	Male	1.4	0.4	1.2	0.6	0.3	Yes
	Female	1.5	0.4	1.5	0.9	0.3	Yes
70–74	Male	2.1	0.3	2.0	0.1	0.1	Yes
	Female	3.0	0.4	3.2	0.8	0.1	Yes
75–79	Male	4.3	0.5	4.0	0.6	0.1	Yes
	Female	5.2	1.3	5.0	1.4	0.2	Yes
80+	Male	9.4	1.5	10.0	3.0	0.2	Yes
	Female	12.3	4.7	11.0	5.0	0.4	Yes

#### Total prevalence

Following the second round, the average estimated prevalence for males was 2.4% (95% CI = 1.9 to 3.0), and 3.1% (95% CI = 2.0 to 4.2) for females. The total estimate of dementia prevalence in the over 60s in India was 2.8% (95% CI = 1.9 to 3.6).

#### Severity

As consensus was achieved for estimates of mild and moderate severity in the first round, the emphasis was on determining whether consensus could be achieved for estimating the percentage of people currently living with severe dementia. In the second round, estimates of dementia severity remained largely unchanged, with the mean estimate of severe dementia was 21.3% (SD = 14.4, Mdn = 17.5, IQR = 18.8, CoV = 0.7). Consensus for this field was not achieved.

## Discussion

There are considerable gaps in evidence with respect to dementia prevalence in India. Among available evidence, there are limited data to allow prevalence estimates at a granular level. This study presented dementia prevalence estimates split by age and sex, but also the proportion of older adults potentially living at different stages of the condition in India. This was achieved using the Delphi process, through generating consensus among experts with substantial experience in epidemiology, neurology and public health in India. These prevalence estimates can aid in understanding the economic impact of dementia to society and further planning for appropriate resource allocation towards care services for dementia in India.

Our consensus estimates generated a dementia prevalence of 2.8% (95% CI = 1.9 to 3.6) for those aged 60 years and above in India. This would equate to approximately 3.9 million people living with dementia in India, when based on population projections for older adults living in India in 2021 (National Commission on Population, 2020). One systematic review estimated the dementia prevalence to be 2% in India^1^ (Choudhary et al., 2021). Despite high quality synthesis, we should be cautious not to assume that such estimates are definitive. For example a recent logistic model for dementia status from LASI, which reported a prevalence of 7.4% for those aged 60 years and above in India (N = 28,949) (Lee et al., 2023). Such variability could be attributed to differences in demographics, region studied, methodology and diagnostic criteria employed (Lee et al., 2023; Ravindranath & Sundarakumar, 2021). The benefit of the Delphi process is that it allows for experts to factor this variability and recognise methodology that might underestimate the true prevalence of dementia.

Furthermore, this Delphi study sought to determine age and sex-specific prevalence estimates for dementia in India. Consensus was not achieved for the prevalence estimate of females aged 60–64. However, in line with previous studies (GBD 2019 Dementia Forecasting Collaborators, 2022; Prince et al., 2013), our consensus estimates demonstrated higher prevalence of dementia in women than in men (3.1% vs 2.4). This may be partially attributed to longer life expectancy in women (Mielke, 2018), or poorer performance on cognitive tasks introduced through lower education attainment in women (Farrell et al., 2020; Zhang, 2006). Furthermore, in comparison to the GBD study (Singh et al., 2021), our consensus estimates were generally within the 95% confidence intervals reported, with only three estimates outside: males between 60 and 64 years of age, males between 65 and 69 years of age, and females aged 80 plus. Therefore, this strengthens the likelihood of our findings being tenable.

In addition to determining dementia prevalence split by age and sex, we also attempted to gain expert consensus on the proportion of those living at different stages of dementia in India. Severity of dementia is known to have a profound impact on estimating costs of care, with a prior study identifying an increase in dementia severity to contribute to a reduction in the proportion of medical costs, but an increase in care-related costs in India (Rao & Bharath, 2013). While no large epidemiological studies have examined dementia severity in the Indian population, a few studies conducted in specific regions of the country offer some insights (Banerjee et al., 2017; Rodriguez et al., 2008). For example, out of the 103 people detected with dementia in Kolkata City, 21.4% had mild, 25.2% had moderate and 53.4% had severe dementia (Banerjee et al., 2017). In our study, the experts estimated that 42.5% of people living with dementia had mild dementia (approximately 1.7 million older adults in India), and 31.9% had moderate dementia (approximately 1.2 million older adults in India). Consensus was not achieved for the percentage of persons living with severe dementia. The inability to achieve consensus in this category, may also be due to absence of relevant data or the use of different datasets by individual experts to estimate severity. For example, one expert broadly based their severity estimates on one study (Saldanha et al, 2010). Overall, understanding how the consensus estimates on dementia severity in India compare to other data is difficult, not least because there are no equivalent data existing in India. In the UK, a similar Delphi consensus processes estimated that from people with late-onset dementia, 55.4% have mild dementia and 32.1% have moderate dementia (Prince et al., 2014). In the US population-based Framingham Heart Study, researchers estimated that 50.4% of Alzheimer’s disease participants had mild severity and 30.3% had moderate severity (Yuan et al., 2021). There is a need for further research in India to determine severity profile of dementia and allow for experts to more accurately estimate severity.

One of the key strengths of this study is that it is the first to use the Delphi method to estimate dementia prevalence and severity in India. The range of experts that have contributed to this study (including prominent neurologists, experienced in epidemiological research and diagnostic tools in India) has provided some validity to our consensus estimates, which have added to the current evidence base on dementia prevalence and severity. We utilized a priori criteria to define how consensus was achieved, and when the Delphi process should be stopped. However, it should also be acknowledged that there is no current gold standard threshold, and that applying different definitions of consensus could yield different results.

Other limitations are also recognized. The Delphi process did not continue to the third round due the dropout rates between round 1 and 2 exceeding 30%, thus potentially affecting the validity of the findings. Experts that dropped out between rounds tended to have higher prevalence estimates in the first round, and hence it is possible that they did not feel comfortable with altering their score to align with others. Another caveat of the process is that the consensus estimates are based completely on expert opinion, although efforts were made to minimise potential bias. As experts draw conclusions largely based on the existing evidence, possible conclusions are considerably dependent on this limited and variable evidence. The process is also susceptible to cognitive biases such as the bandwagon effect (in which people will change their responses just so they align with others, irrespective of their own belief) and belief perseverance (in which people will not change their beliefs even in light of new information) (Winkler & Moser, 2016). The extent to which the experts were given direction to formulate their estimates, may have also influenced their estimates. For example, we did not provide guidance on the diagnostic criteria that should be used to estimate prevalence, or explicitly define dementia. It is unclear the extent to which this lack of guidance may have shaped experts’ decisions regarding the estimates generated. All experts had backgrounds in medicine, thus potentially minimising the adoption of non-clinical definitions and diagnostic criteria of dementia. As with any Delphi process, the choice of experts is likely to influence the conclusions made, as their input will be based on their training and experiences.

In conclusion, our findings provide detailed information regarding the number of people living with dementia in India and also novel insights into how many people are potentially living at each stage of the condition. Such data can be utilized to estimate the economic impact of dementia and also allow for better allocation of resources towards dementia care in India until further primary evidence is generated.

## Supplemental Material

Supplemental Material - Estimating the number of people living with dementia at different stages of the condition in India: A Delphi process Supplemental Material for Estimating the number of people living with dementia at different stages of the condition in India: A Delphi process by Nicolas Farina, Jayeeta Rajagopalan, Suvarna Alladi, Aliaa Ibnidris, Cleusa P Ferri, Martin Knapp, Adelina Comas-Herrera in Dementia

## Note

1. The meta-analysis was not limited by age and included data from studies that estimated prevalence in those aged ≥ 50 years.
