# Supplementary material for: Estimating the number of people living with dementia at different stages of the condition in India: A Delphi process
Source: Dementia (London). 2023 Jun 5;23(3):438–51. doi: 10.1177/14713012231181627 (PMC11041066; doi:10.1177/14713012231181627)
Supplement: Supplemental Material - Estimating the number of people living with dementia at different stages of the condition in India: A Delphi process [file sj-pdf-1-dem-10.1177_14713012231181627.pdf]

Supplementary Table 1. Brief description of the experts that participated in the Delphi process.

Expert #	Expertise
**1**	Neurologist, been a part of dementia epidemiological studies conducted in the Indian context
**2**	Neurologist, been a part of dementia epidemiological studies conducted in the Indian context
**3**	Physician and Research Professor, been a part of national and international level research on burden of neurological disorders.
**4**	Leads a chapter of an Alzheimer's related NGO in India and is a neurologist.
**5**	Neurologist, has aided in a study validating culturally appropriate diagnostic tools for dementia/MCI
**6**	Neurologist, has aided in a study validating culturally appropriate diagnostic tools for dementia/MCI
**7**	Public health researcher and a neurologist
**8**	Geriatric Psychiatrist and leads a chapter of Alzheimer's related NGO in India
